# Evaluation of Radiosensitization and Cytokine Modulation by Differentially PEGylated Gold Nanoparticles in Glioblastoma Cells

**DOI:** 10.3390/ijms241210032

**Published:** 2023-06-12

**Authors:** Bríanna N. Kerr, Daniel Duffy, Caitríona E. McInerney, Ashton Hutchinson, Inaya Dabaja, Rana Bazzi, Stéphane Roux, Kevin M. Prise, Karl T. Butterworth

**Affiliations:** 1 Patrick G. Johnston Centre for Cancer Research, Queen’s University Belfast, Belfast BT9 7AE, Northern Ireland, UK; bkerr17@qub.ac.uk (B.N.K.);; 2 Institute Utinam, UMR 6213 CNRS-UFC, University of Franche, 25000 Comté, France

**Keywords:** glioblastoma, gold nanoparticles, radiation, radiotherapy, cytokines, radiobiology

## Abstract

Glioblastoma (GBM) is known as the most aggressive type of malignant brain tumour, with an extremely poor prognosis of approximately 12 months following standard-of-care treatment with surgical resection, radiotherapy (RT), and temozolomide treatment. Novel RT-drug combinations are urgently needed to improve patient outcomes. Gold nanoparticles (GNPs) have demonstrated significant preclinical potential as radiosensitizers due to their unique physicochemical properties and their ability to pass the blood–brain barrier. The modification of GNP surface coatings with poly(ethylene) glycol (PEG) confers several therapeutic advantages including immune avoidance and improved cellular localisation. This study aimed to characterise both the radiosensitizing and immunomodulatory properties of differentially PEGylated GNPs in GBM cells in vitro. Two GBM cell lines were used, U-87 MG and U-251 MG. The radiobiological response was evaluated by clonogenic assay, immunofluorescent staining of 53BP1 foci, and flow cytometry. Changes in the cytokine expression levels were quantified by cytokine arrays. PEGylation improved the radiobiological efficacy, with double-strand break induction being identified as an underlying mechanism. PEGylated GNPs also caused the greatest boost in RT immunogenicity, with radiosensitization correlating with a greater upregulation of inflammatory cytokines. These findings demonstrate the radiosensitizing and immunostimulatory potential of ID11 and ID12 as candidates for RT-drug combination in future GBM preclinical investigations.

## 1. Introduction

Glioblastoma multiforme (GBM) is the most commonly occurring malignant primary brain tumour, representing 77–81% of all primary malignant tumours of the central nervous system (CNS) [1]. GBM is a high-grade glioma which corresponds to the grade IV classification of CNS tumours from the World Health Organization (WHO) [2]. Despite advances in understanding the molecular characterisation of GBM, it carries the worst prognosis of all solid tumours, a median overall survival of 12 months [3]. The current standard of care for GBM involves surgical resection followed by chemo-radiotherapy (RT) with temozolomide (TMZ) and adjuvant TMZ [4]. However, these have limited efficacy and are rarely curative; therefore, there remains an urgent, unmet clinical need for the development of novel treatment strategies to improve GBM outcomes.

A potential therapeutic approach is to improve the efficacy of post-operative RT in the management of GBM by combining RT with novel radiosensitizers [5]. Over the past two decades, there has been continued interest in the development of gold nanoparticles (GNPs) as radiosensitizers [6,7,8,9]. These applications are based on the unique physio-chemical properties of high atomic number (Z) materials such as gold (Au, Z = 79), which include the ability to preferentially absorb X-rays compared to soft tissues, innate image contrast enhancement, and passive targeting through the enhanced permeability retention (EPR) effect [10,11]. Also, GNPs can pass freely through the blood–brain barrier (BBB) to improve bioavailability and can be easily modified to optimise functionality, including size, surface charge, and surface coatings [12,13].

A wide range of different GNPs have been explored preclinically, supporting their potential use as radiosensitizers in GBM. In these studies, poly(ethylene) glycol (PEG) is often functionalised on the GNP surface to improve cellular biodistribution and facilitate immune escape to increase the blood circulation time and localisation in targeted tumour sites [14,15]. However, the optimum NP formulation for radiosensitization is still under debate [16]. Therefore, this study aimed to evaluate the radiosensitizing effects of three different GNPs coated with a macrocyclic chelator (DOTAGA) for the immobilization of ions of interest for medical imaging functionalized by thioctic acid (TA) to ensure stable anchoring onto the gold cores. The NPs were synthesized by the reduction of gold salt with sodium borohydride in the presence of TADOTAGA, TAPEG_4_DOTAGA, or TAPEG_11_DOTAGA and were designated as ID10, ID11, and ID12, respectively. Each of the NPs had a similar core size, between 2 and 3 nm, and similar hydrodynamic diameters for ID10 and ID11 (6.4 nm ± 2.3 and 1.9) but larger diameters for ID12 (8.3 ± 1.7 nm) (Table A1). The particles were differentially PEGylated with 0, 4, and 11 oxyethylene units in the PEG chain inserted between the TA and DOTAGA for ID10, ID11, and ID12, respectively (Figure A2).

In addition, there remains a gap in the current understanding of the impacts of GNPs on the immune system. This is of particular importance given the success of immune-oncology strategies that are becoming increasingly established in clinics across a range of indications including resistant GBM tumours [17]. GBM is considered one of the most immune “cold” tumours; thus, therapeutic approaches that stimulate an anti-tumour immune response are highly desirable to improve the efficacy of RT [18]. One such therapy includes immune checkpoint modulators, which have gained recent attention in the context of GBM, with a current phase II clinical trial investigating the effect of TMZ followed by ipilimumab, which targets PD-1 and CTLA-4, and nivolumab in recurrent GBM patients (NCT04145115) [19]. Other therapies that are currently under clinical investigation are chimeric antigen receptor (CAR) T cell therapies and cancer vaccines, with the latter being studied in newly diagnosed GBM patients (NCT00639639) [20,21].

The immunomodulatory effects of RT have been well characterised and shown to be both immuno-stimulatory and immuno-suppressive within the tumour microenvironment [22,23]. However, the immunomodulatory impacts of different GNPs are unknown. Thus, this study also aimed to investigate the impact of differentially PEGylated GNPs and radiation as both monotherapies and combination treatments on cytokine modulation in GBM cells in vitro. Cytokines are important small molecule signalling proteins which influence various tumour-associated processes such as apoptosis, angiogenesis, inflammation, and cell differentiation and growth [24]. For this preliminary body of work, human cytokine antibody arrays were performed to simultaneously evaluate the expression of 80 human cytokines following each treatment in two GBM cell lines. Upon analysis of the cytokine expression levels, differentially expressed cytokines and their corresponding genes were used to identify significantly associated Gene Ontology (GO) biological pathways, via bioinformatics analysis, from which comparisons can be drawn between each treatment.

As this is a novel topic which has not yet been explored, this research could provide greater insight into the effect of different GNP formulations and ionising radiation, as monotherapies or combination treatments, on immune activation in GBM cells. This potentially presents an opportunity to optimise treatments and thereby maximise tumour control for GBM patients in the future.

## 2. Results

### 2.1. Evaluation of the Effects of GNPs on Cell Survival

**Figure 1:**
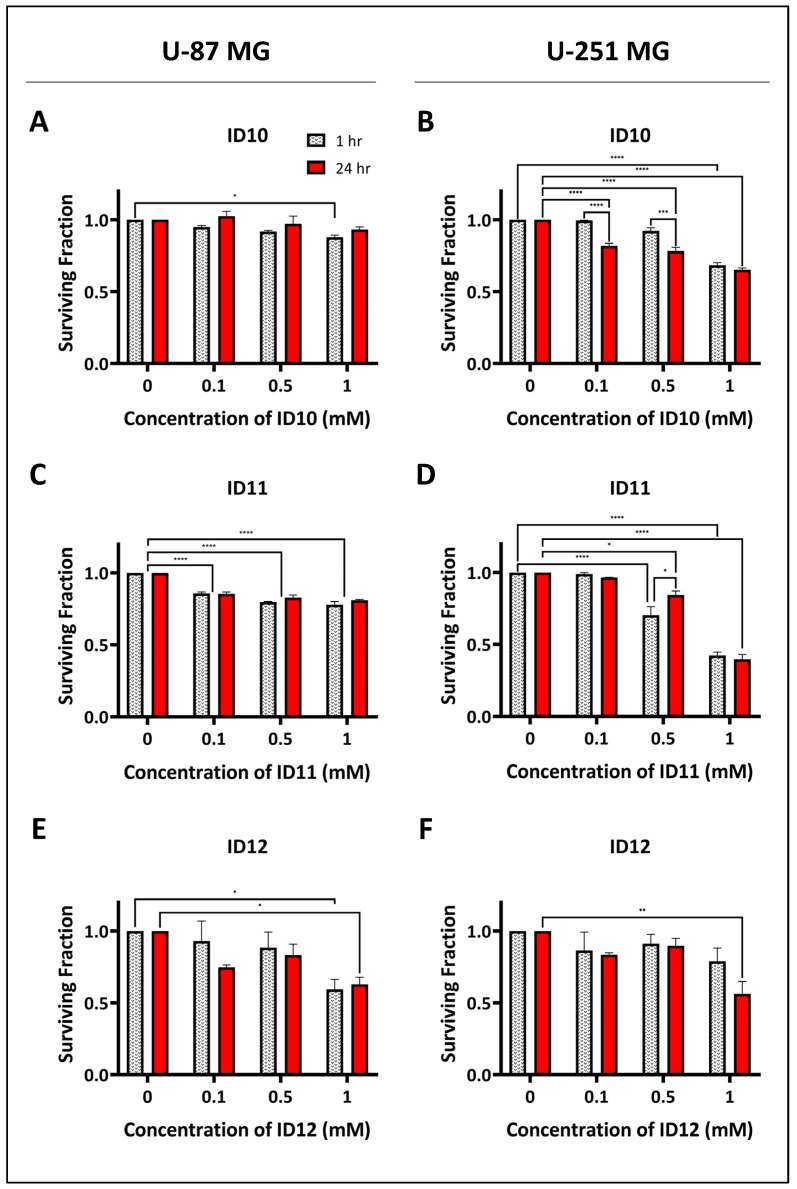
Evaluation of the cytotoxic effects of differentially PEGylated GNPs on GBM cell models. Clonogenic assays were performed in the absence of radiation. Treatment with ID10 in (**A**) U-87 MG and (**B**) U-251 MG cells; treatment with ID11 in (**C**) U-87 MG and (**D**) U-251 MG cells; treatment with ID12 in (**E**) U-87 MG and (**F**) U-251 MG cells. Cells were transiently treated with all GNPs at 0.1 mM, 0.5 mM, and 1 mM for 1 h or 24 h. ID10 has 0 PEG groups, ID11 has 4 PEG groups, and ID12 has 11 PEG groups. Bars are presented as means ± SEM. Statistical significances are represented as *p* < 0.05 = *; *p* < 0.01 = **; *p* < 0.001 = ***; and *p* < 0.0001 = ****. Experiments were performed in triplicate (n = 3).

Clonogenic survival assays were used to assess the colony formation abilities of U-87 MG and U-251 MG cells. Cell survival was measured in response to 1 h and 24 h exposure of the three tested GNPs, ID10, ID11, and ID12, as shown in Figure 1. Treatment of the U-87 MG cells with the unPEGylated GNP ID10 at 1 mM caused a small but significant decrease in cell survival following 1 h exposure at 87% survival compared to the untreated control (*p* = 0.0413; Figure 1A). This indicates both a time- and dose-dependent response to ID10. Similarly, 1 h treatment of U-251 MG cells with 1 mM ID10 also caused significant cytotoxicity at 68% survival (*p* = < 0.0001; Figure 1B). In addition, 24 h treatment with ID10 at all of the tested concentrations (0.1–1 mM) caused a significant decrease in U-251 MG cell survival, indicating a time-dependent response.

Figure 1C shows that exposure to 0.1 mM (85% and 85%), 0.5 mM (79% and 82%) and 1 mM (77% and 80%) 1 h and 24 h, respectively, had significant cytotoxic effects on U-87 MG cells (all *p*-values are <0.0001). Therefore, the high levels of ID11-mediated cytotoxicity were not concentration- or time-dependent. The treatment of U-251 MG cells with ID11 for 1 h at 0.5 and 1 mM, as seen in Figure 1D, caused a significant decrease in cell survival at 70% and 42%, respectively (both *p*-values are <0.0001). Similar results were observed following 24 h exposure to 0.1 and 0.5 mM ID11 at 84% (*p* = 0.0173) and 39% (*p* = <0.0001) survival, respectively.

Lastly, only treatment with 1 mM ID12 for both 1 h and 24 h resulted in significant cytotoxicity in U-87 MG cells, with survival at 59% (*p* = 0.0243) and 62% (*p* = 0.0462), respectively (Figure 1E). Figure 1F shows that only 24 h treatment with 1 mM ID12 caused a significant decrease in U-251 MG cell survival at 56% (*p* = 0.008). Therefore, the data indicate that ID12 exposure time and concentration contributed to the observed U-87 MG and U-251 MG cytotoxicity. Also, the varying cytotoxic effects observed between the differentially PEGylated GNPs allude to the potential impact of PEG on GBM cell survival.

### 2.2. Impact on Clonogenic Cell Survival

**Figure 2:**
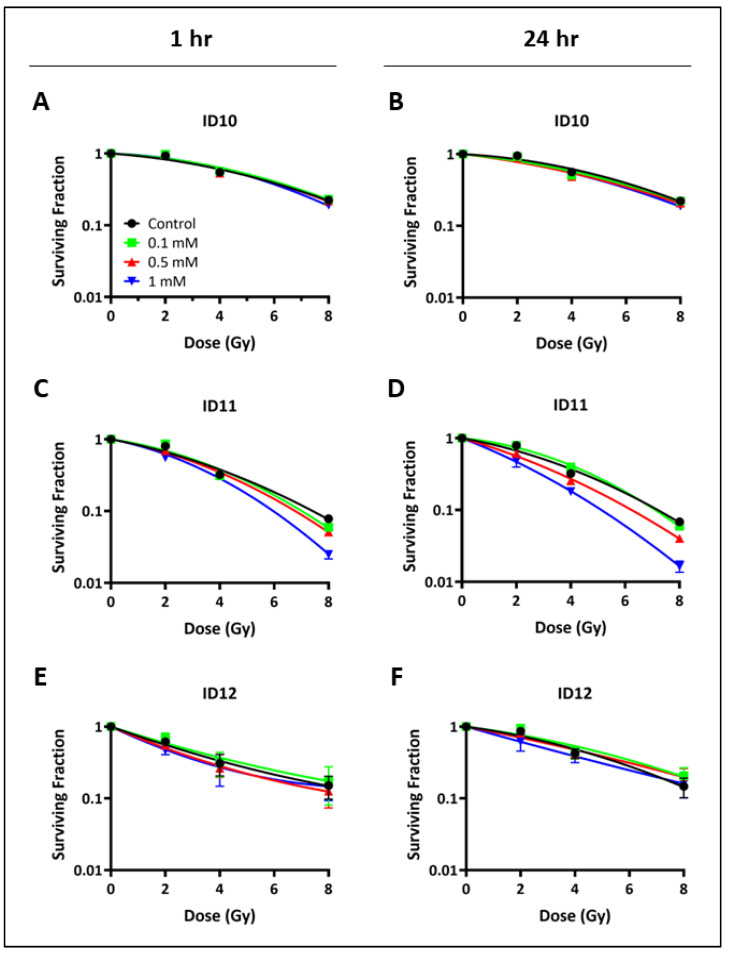
Impact of differentially PEGylated GNPs on clonogenic cell survival following transient exposure in U-87 MG cells. Clonogenic assays were performed. U-87 MG cells were treated with ID10 for (**A**) 1 h and (**B**) 24 h, with ID11 for (**C**) 1 h and (**D**) 24 h, and with ID12 for (**E**) 1 h and (**F**) 24 h, prior to irradiation with 2–8 Gy of X-rays. Cells were transiently treated with all GNPs at 0.1 mM, 0.5 mM, and 1 mM. Graphs are presented as means ± SEM. Experiments were performed in triplicate (n = 3).

**Figure 3:**
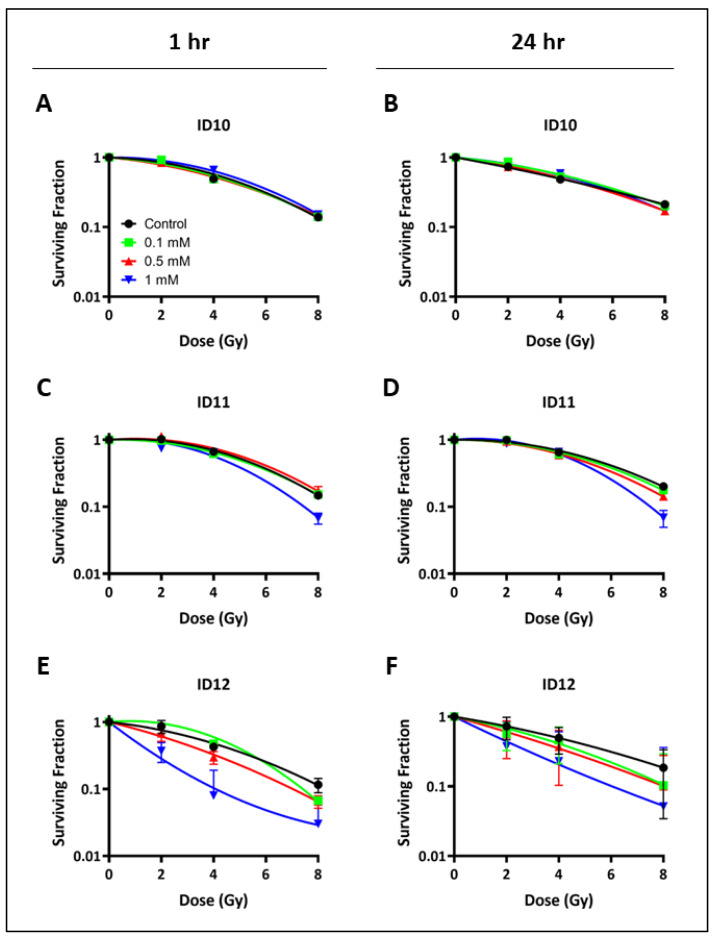
Impact of differentially PEGylated GNPs on clonogenic cell survival following transient exposure in U-251 MG cells. Clonogenic assays were performed. U-251 MG cells were treated with ID10 for (**A**) 1 h and (**B**) 24 h, with ID11 for (**C**) 1 h and (**D**) 24 h, and with ID12 for (**E**) 1 h and (**F**) 24 h, prior to irradiation with 2–8 Gy of X-rays. Cells were transiently treated with all GNPs at 0.1 mM, 0.5 mM, and 1 mM. Graphs are presented as means ± SEM. Experiments were performed in triplicate (n = 3).

Clonogenic survival assays were used to assess the colony formation abilities of U-87 MG and U-251 MG cells. Cell survival was measured in response to GNP treatments for 1 h and 24 h prior to irradiation. The dose–response curves in Figure 2 and Figure 3 show the cell survival data for U-87 MG and U-251 MG cells, respectively. A summary of the radiobiological parameters is presented in Table 1, including the α/β ratios (defined as the ratio of the α and β parameters derived from the linear quadratic (LQ) model, Section 4.3), SF2 values (defined as the surviving fraction at 2 Gy), and sensitizer enhancement ratios (SER, defined as the ratio of the mean inactivation dose for control and NP-treated cells, Section 4.3).

Treatment with 1 mM ID11 for 1 h (Figure 2C) and 24 h (Figure 2D) prior to irradiation had a clear radiosensitizing effect on U-87 MG cells with SER values of 1.21 and 1.45, respectively. Similar radiosensitization was observed following 1 h (Figure 2E) and 24 h (Figure 2F) of treatment with 1 mM ID12 with SER values of 1.13 and 1.16, respectively. In contrast, no impact on U-87 MG cell survival was observed following transient treatment with ID10, the unPEGylated GNP, with SER values for 1 mM and 1 h (Figure 2E) and 24 h (Figure 2F) exposure of 0.98 and 1.09, respectively.

Similarly, Figure 3 shows that treatment with 1 mM ID11 for 1 h (Figure 3C) and 24 h (Figure 3D) prior to irradiation had a clear radiosensitizing effect on U-251 MG cells with SER values of 1.17 and 1.10, respectively. Similar radiosensitization was observed following 1 h (Figure 3E) and 24 h (Figure 3F) of treatment with 1 mM ID12 with SER values of 2.08 and 1.70, respectively. In contrast, no impact on U-251 MG cell survival was observed following transient treatment with ID10, the unPEGylated GNP, with SER values for 1 mM and 1 h (Figure 3E) and 24 h (Figure 3F) exposure of 0.96 and 0.95, respectively.

### 2.3. Evaluation of Double-Strand Break Induction and Repair

**Figure 4:**
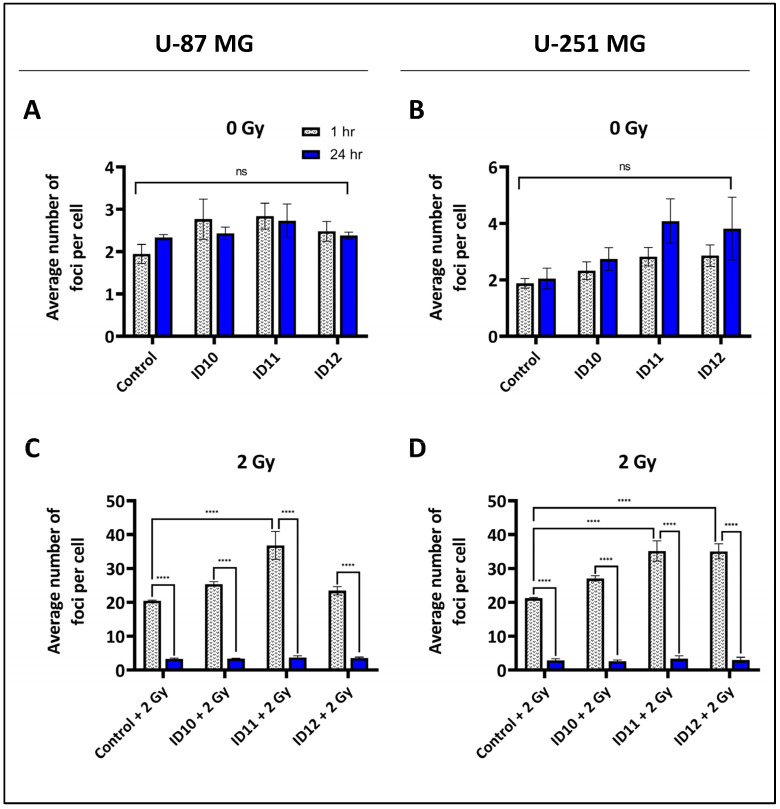
Induction of DSB foci by differentially PEGylated GNPs alone and in combination with RT in GBM cell models. DNA DSBs were quantified by immunofluorescent staining of 53BP1. (**A**) U-87 MG cells and (**B**) U-251 MG cells were treated with 1 mM ID10, ID11, and ID12 for 1 h, then fixed after 1 h and 24 h. (**C**) U-87 MG and (**D**) U-251 MG cells were treated with 1 mM ID10, ID11, and ID12 for 1 h prior to 2 Gy irradiation with X-rays then fixed at 1 h and 24 h post-irradiation. Bars are presented as means ± SEM. Statistical significances are represented as *p* < 0.0001 = ****; ns = not significant. Experiments were performed in triplicate (n = 3).

The number of double-strand breaks (DSBs) foci per cell was quantified by performing immunofluorescent staining of 53BP1. Both U-87 MG and U-251 MG cells were treated with 1 mM ID10, ID11, and ID12 for 1 h prior to 0 Gy or 2 Gy irradiation and then fixed after 1 h and 24 h, as shown in Figure 4. Transient treatment of both U-87 MG (Figure 4A) and U-251 MG (Figure 4B) cells with each GNP had a non-significant impact on DSB foci levels in the absence of radiation compared to the respective untreated control levels at both time points.

At 1 h and in the presence of 2 Gy irradiation, ID11 was the only GNP to significantly increase DNA damage, with 36.82 foci per cell compared to the irradiated control (20.49) in U-87 MG cells (Figure 4C). For U-251 MG cells, both ID11 plus 2 Gy (35.17) and ID12 plus 2 Gy (35.06) significantly increased the number of DSB foci at 1 h post-irradiation compared to the irradiated control (21.24) (Figure 4D). In both cell lines, all GNP plus 2 Gy combination treatments and the irradiated control had a significant difference in foci number per cell between the respective 1 h and 24 h time points, suggesting high levels of DNA damage repair occurred independently of treatment. All significant results were *p* = <0.0001.

### 2.4. Evaluation of Cell Cycle Effects

Flow cytometry was performed to quantify the percentage of GBM cell populations in each phase of the cell cycle (Sub G1, G1, S, and G2/M) following 1 h treatment with 1 mM of each GNP prior to 2 Gy irradiation and for unirradiated cells. All samples were fixed at 72 h post-treatment or post-irradiation.

**Figure 5:**
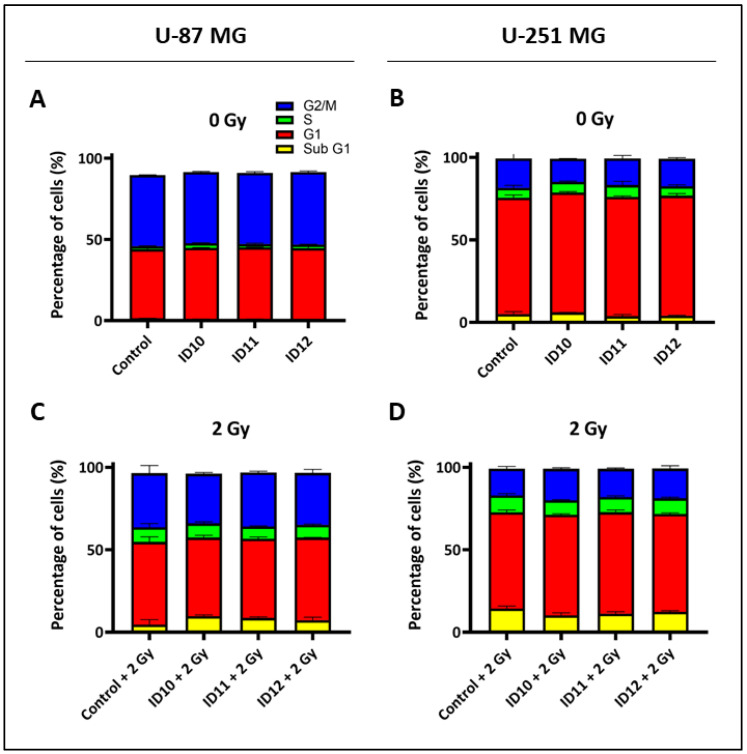
Impact of differentially PEGylated GNPs on cell cycle alone and in combination with RT in GBM cell models. Flow cytometry was performed to assess cell cycle distribution. (**A**) U-87 MG and (**B**) U-251 MG cells were treated with 1 mM ID10, ID11 and ID12 for 1 h then fixed after 72 h. (**C**) U-87 MG and (**D**) U-251 MG cells were treated with 1 mM ID10, ID11, and ID12 for 1 h prior to 2 Gy irradiation with X-rays then fixed at 72 h post-irradiation. Bars are presented as means ± SEM. Experiments A, C, and D are n = 3, and B is n = 2.

The bar graphs show that transient treatment of both U-87 MG (Figure 5A) and U-251 MG (Figure 5B) cells with each GNP in the absence of radiation had a non-significant impact on Sub G1, G1, S, and G2/M arrest compared to the untreated control. Similar non-significant results were also observed with the combination of each GNP plus 2 Gy irradiation in the U-87 MG (Figure 5C) and U-251 MG (Figure 5D) cells compared to the respective irradiated controls. Thus, all GNPs and 2 Gy, as monotherapies and in combination with RT, have minimal impacts on cell GBM cell cycle arrest at 72 h.

### 2.5. Comparison of Overall Cytokine Profiles and Differentially Expressed Cytokines

**Figure 6:**
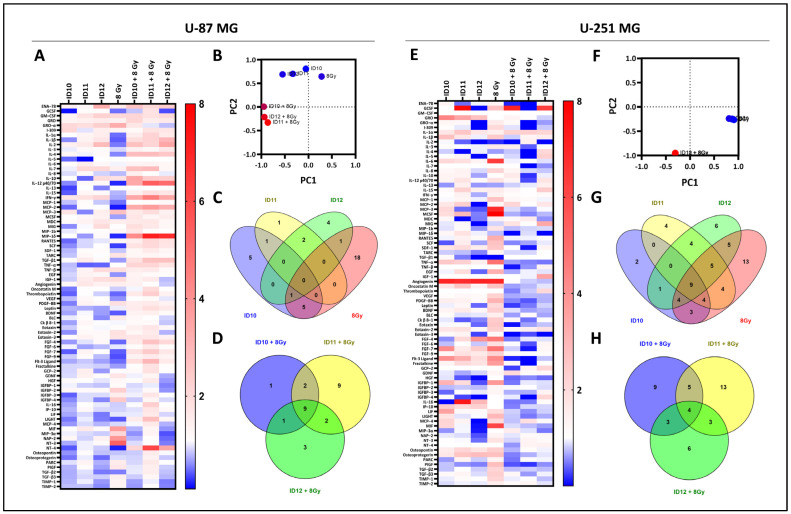
Overall cytokine expression profiles and comparison of differentially expressed cytokines following treatment with GNPs alone and in combination with RT in GBM cell models. (**A**) A heatmap representing the overall cytokine profile for U-87 MG cells following GNPs and 8 Gy treatment as monotherapies and in combination with RT. (**B**) A principal component analysis plot comparing the potential similarities of all treatments in U-87 MG cells. Venn diagrams (**C**,**D**) compare differentially expressed cytokines with fold changes of <0.5 and >2 compared to control expression levels in U-87 MG cells for monotherapies and combination treatments, respectively. (**E**) A heatmap representing the overall cytokine profile for U-251 MG cells following GNPs and 8 Gy treatment as monotherapies and in combination. (**F**) A principal component analysis plot comparing the potential similarities of all treatments in U-251 MG cells. Venn diagrams (**G**,**H**) compare differentially expressed cytokines with fold changes of <0.5 and >2 compared to control expression levels in U-251 MG cells for monotherapies and combination treatments, respectively.

The expression of inflammatory cytokines following treatment with differentially PEGylated GNPs and 8 Gy RT as monotherapies and in combination with RT was analysed using human cytokine antibody arrays in U-87 MG and U-251 MG cells. The heatmap in Figure 6A presents the overall fold changes in expression for 80 different cytokines following treatment with all three GNPs and 8 Gy as monotherapies and combination treatments compared to the unirradiated control for monotherapies, and the irradiated control for combination treatments in U-87 MG cells. For the monotherapies, similar cytokine profiles were observed, suggesting a conserved cytokine response. Similar profiles were also observed for the GNP and 8 Gy combination treatments. However, ID11 plus 8 Gy appears to have a greater immunogenic effect, with the pronounced upregulation of MIP-1δ (7.13-fold) and NT-4 (6.08-fold).

The principal component analysis (PCA) plot in Figure 6B which compares the overall cytokine expression changes for all of the treatments illustrates two distinct clusters, the monotherapies (blue) and the GNP-RT combination treatments (red), which is indicative of intra-treatment similarities.

**Figure A1:**
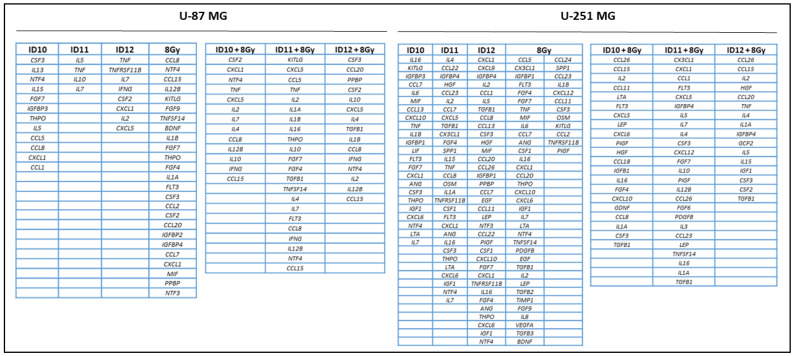
Summary of the gene names corresponding to the differentially expressed cytokines in response to each treatment in U-87 MG and U-251 MG cells. The comparisons of these data are presented as Venn diagrams in Figure 6.

**Figure A2:**
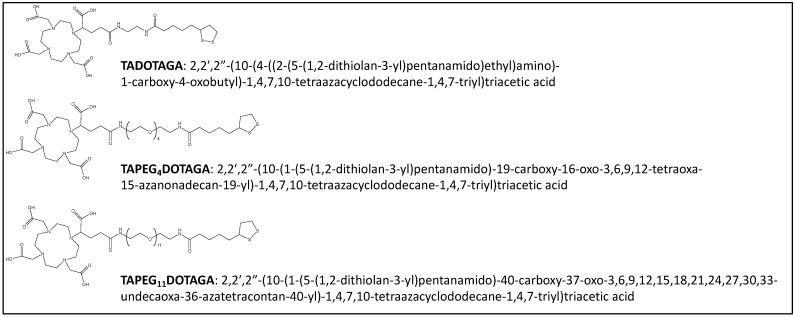
The chemical structures and names of TADOTAGA, TAPEG_4_DOTAGA, and TAPEG_11_DOTAGA.

The Venn diagrams in Figure 6C,D show the shared or unique differentially expressed cytokines between each monotherapy and combination treatment with RT, respectively, in U-87 MG cells (Figure A1). Interestingly, there were no differentially expressed cytokines that were common to all three GNP monotherapy treatments, and two cytokines were shared between the PEGylated GNPs ID11 and ID12, specifically TNF-α and IL-7. Similar fold changes were observed for TNF-a at 0.42 and 0.44, and IL-7 at 2.06 and 2.07 following treatment with ID11 and ID12, respectively. These data suggest a potential role of PEG in mediating cytokine expression. In addition, irradiation with 8 Gy resulted in the highest number of differently expressed cytokines [18] that were unique to one treatment, creating a radiation-specific cytokine profile.

For combination treatments with RT, nine differentially expressed cytokines were shared between all three GNP-RT treatments, and there were two in common between the PEGylated GNP-RT treatments (IL-1β and TGF-β1). The combination treatment resulting in the highest number of unshared differentially expressed cytokines was ID11 plus 8 Gy, with nine unique cytokines (SCF, RANTES, IL-1α, IL-16, Thrombopoietin, FGF-7, FGF-4, LIGHT, and Flt-3 Ligand).

Similar to Figure 6A, heatmap E shows the overall fold changes in expression for 80 different cytokines following treatment with all three GNPs and 8 Gy as monotherapies and combination treatments compared to the unirradiated control for the monotherapies, and the irradiated control for the combination treatments in U-251 MG cells. For the monotherapy treatments, ID10 and ID11 had similar cytokine profiles, with many upregulated and downregulated cytokines, compared to ID12 and 8 Gy, which predominantly downregulated and upregulated cytokines, respectively.

For combination treatments, heatmap E also shows that there are many similar fold changes seen between each of the treatments for U-251 MG cells compared to the 8 Gy control, indicating somewhat of an overall conserved effect on the secreted cytokines. Both ID10 plus 8 Gy and ID11 plus 8 Gy had a predominantly downregulatory effect on cytokine expression compared to the 8 Gy control. In contrast, ID12 plus 8 Gy downregulated and upregulated many cytokines. Two examples of ID12 plus 8 Gy achieving higher upregulation compared to the other combinations are MIG (3.36-fold) and IGF-1 (3.49-fold). The anti-inflammatory TGF-β1 exhibits the highest-fold change for all combinations of ID10 plus 8 Gy (2262.20-fold), ID11 plus 8 Gy (772.79-fold), and ID12 plus 8 Gy (415.37-fold), suggesting a high inflammatory response.

The PCA plot displayed in Figure 6F compares the overall cytokine expression changes for all of the treatments and, similarly to the U-87 MG data, it illustrates two distinct clusters, the monotherapies (blue) and the GNP-RT combination treatments (red), which is indicative of intra-treatment similarities.

The Venn diagrams in Figure 6G,H show the shared or unique differentially expressed cytokines between each monotherapy and combination treatment, respectively, in U-251 MG cells (Figure A1). The comparison of monotherapies in Figure 6G shows that nine cytokines are differentially expressed in all four arrays (IL-16, MCP-3, GRO-α, Angiogenin, GCSF, Thrombopoietin, IGF-1, GCP-2, and NT-4). Four proteins are differentially expressed in response to the PEGylated GNPs ID11 and ID12 (MDC, IGFBP-4, HGF, and MCP-2), suggesting a potential role for PEG in mediating cytokine expression. Of particular interest is that no cytokines were commonly expressed following treatment with the GNPs. The highest number of differentially expressed cytokines unique to one treatment was observed following 8 Gy RT, with 13 cytokines being specific to a radiation cytokine profile. The comparison of GNP-RT treatments in Figure 6H shows that only four differentially expressed cytokines are conserved between all three of the combination treatments (GCSF, Eotaxin-3, IL-1α, and TGF-β1). Three proteins are in common between the two PEGylated GNP combinations (IGFBP-4, IL-5, and IL-4), suggesting that changes in the PEG chain length produce a varied immunogenic response.

### 2.6. Pathway Enrichment Analysis

To further understand the biological pathways enriched in GBM cells following GNP and 8 Gy RT treatment as monotherapies and also in combination, Gene Ontology (GO) pathway enrichment analysis was performed. To achieve this, the differentially expressed cytokines identified in each array were transformed into their corresponding gene names, and the associated processes were identified.

**Figure 7:**
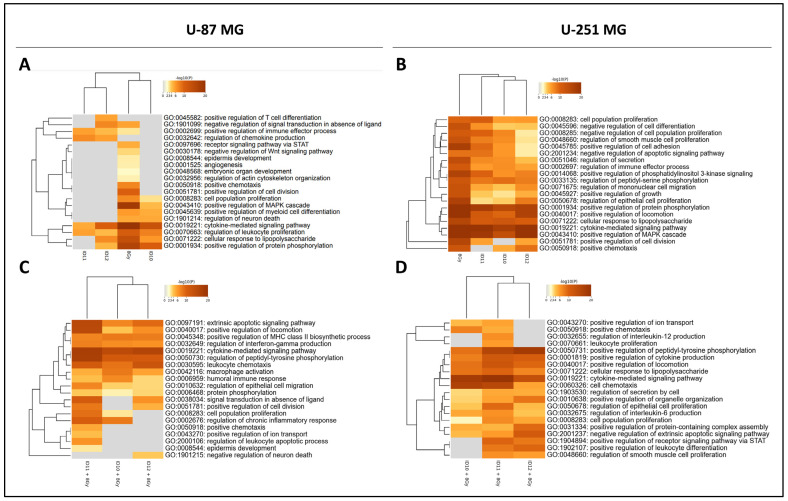
Analysis of enriched Gene Ontology biological processes following transient treatment with differentially PEGylated GNPs and 8 Gy as monotherapies and in combination in GBM cell models. Heatmaps (**A**,**B**) show GO-enriched biological processes in U-87 MG and U-251 MG cells, respectively, following GNP and 8 Gy monotherapy treatments. Heatmaps (**C**,**D**) show GO-enriched biological processes in U-87 MG and U-251 MG cells, respectively, following treatment with GNPs and 8 Gy in combination, respectively. Dark brown boxes correlate with higher numbers of genes associated with the biological process and vice versa. Grey boxes mean no genes are associated with the biological process.

The comparison of enriched GO processes following monotherapy treatment in U-87 MG cells (Figure 7A) shows that 8 Gy RT resulted in the most enriched processes due to having the highest number of differentially expressed cytokines, including the receptor signalling pathway via STAT, angiogenesis, negative regulation of the Wnt signalling pathway, and positive regulation of cell division and chemotaxis. Also, the enrichment of cell population proliferation, the regulation of leukocyte proliferation, and the positive regulation of MAPK cascade and protein phosphorylation processes were the highest for irradiation only compared to the GNP-treated samples. Treatment with the PEGylated GNPs ID11 and ID12 resulted in the highest enrichment for the regulation of chemokine production, and the positive regulation of the immune effector process was highest in ID11. Treatment with ID12 showed enrichment for the positive regulation of T-cell differentiation.

The comparison of enriched GO processes following monotherapy treatment in U-251 MG cells (Figure 7B) shows that the enrichment of cell population proliferation, negative regulation of cell differentiation, and positive regulation of the phosphatidylinositol 3-kinase (PI3K) signalling is the highest in response to both 8 Gy and ID11. Negative regulation of the cell population and apoptotic signalling pathways, alongside positive regulation of cell adhesion, was highly enriched for 8 Gy, ID10, and ID11. Treatment with 8 Gy and ID12 resulted in the highest enrichment of regulation of mononuclear cell migration, epithelial cell proliferation, and positive regulation of growth. All of the treatments except ID11 were highly enriched for positive chemotaxis. Furthermore, all of the treatments resulted in high levels of enrichment for positive regulation of MAPK cascade.

For GNP-RT combination treatments in U-87 MG cells (Figure 7C), the comparison of enriched GO processes shows that ID11 plus 8 Gy had enrichment for most processes. The processes highly enriched by this combination include the extrinsic apoptotic signalling pathway, positive regulation of locomotion and cell division, regulation of peptidyl-kinase phosphorylation, epithelial cell migration and chronic inflammation, and cell population proliferation. The processes which were exclusively impacted by ID11 plus 8 Gy only are the regulation of the leukocyte apoptotic process and the positive regulation of ion transport. Furthermore, macrophage activation and humoral immune response were the most enriched with ID10 plus 8 Gy, and leukocyte chemotaxis with both ID11 plus 8 Gy and ID12 plus 8 Gy. Interestingly, interferon-γ production was similarly enriched across all three arrays, with ID11 plus 8 Gy achieving a slightly higher response.

A comparison of the GO-enriched biological processes following GNP-RT combinations in U-251 MG cells (Figure 7D) showed that combined irradiation at 8 Gy with ID11 and ID12 caused higher enrichment for most processes in comparison to ID10 plus 8 Gy. These 2 types of PEGylated GNP in combination with radiation caused the highest enrichment for the positive regulation of peptidyl-kinase phosphorylation, receptor signalling via STAT, and leukocyte differentiation. Treatment with ID10 plus 8 Gy and ID11 plus 8 Gy had the greatest impact on cell chemotaxis. Moreover, only ID11 plus 8 Gy enriched leukocyte proliferation and it caused the highest enrichment for the positive regulation of epithelial cell proliferation and cell population proliferation. The combination of ID12 plus 8 Gy had the greatest impact on the positive regulation of protein-containing complex assembly and the negative regulation of the extrinsic apoptotic signalling pathway. Several processes had similar enrichment levels across all three of the treatments, including the positive regulation of cytokine production and locomotion, the cellular response to lipopolysaccharide, and the cytokine-mediated signalling pathway, potentially highlighting a conserved immunogenic effect of GNPs, regardless of PEGylation status, in combination with radiation.

## 3. Discussion

This study, first, aimed to assess the radiosensitizing potential of differentially PEGylated GNPs in combination with kVp X-rays in two GBM cell lines in vitro to better understand the relationship between PEG chain length and radiation response. The augmentation of GNP surface coatings with PEG (PEGylation) is known to confer many benefits in the context of delivery to target sites. The presence of PEG reduces the number of potential binding sites for opsonins, which typically tag a foreign species for phagocytosis by macrophages [25]. Escaping macrophage uptake beneficially improves GNP blood circulation time and thereby improves delivery to target sites such as tumours [26,27]. In addition, PEGylation is also known to reduce the number of protein interactions during circulation to favourably lower GNP aggregation and liver accumulation, and, ultimately, achieve elimination [28]. Therefore, we hypothesise that the PEGylated GNPs, potentially by reduced opsonisation, have enhanced stability and improved cellular uptake. These properties may potentiate the radiosensitization of GBM cell models compared to the unPEGylated GNP analogue, yet require further experimental validation.

Firstly, in the absence of radiation, the unPEGylated GNP ID10 had a small but still significant cytotoxic effect at the highest concentration which was tested (1 mM), following 1 h incubation in both U-87 MG and U-251 MG cells. The lack of cytotoxicity observed after 24 h treatment with ID10 in U-87 MG cells was also reported by Salvanou et al. at a comparable concentration [29]. However, the significant decrease in cell survival seen in this study following 1 h treatment with 1 mM ID10 in both cell lines correlated with non-significant increases in DNA DSBs, suggesting the decreased cell survival in response to ID10 was not caused by DSB formation. When combined with radiation, our data clearly demonstrate that ID10 did not sensitize both U-87 MG and U-251 MG cells at both exposure times. This lack of radiosensitivity induced by ID10 also correlated with a non-significant effect on the DNA damage yield in combination with 2 Gy in both cell lines. Thus, our experimental findings indicate the unPEGylated GNP had minimal potential as a radiosensitizer when combined with RT in both tested cell lines.

The second GNP that we evaluated was ID11, which contained four PEG groups within each PEG chain in the surface coating, and which, to our knowledge, has not yet been previously explored in the literature. ID11 caused significant cytotoxicity at all of the tested concentrations in the U-87 MG cells and higher concentrations in the U-251 MG cells. However, this did not correlate with any significant alterations in the DNA damage levels, suggesting that reductions in cell survival due to ID11 treatment were independent of DSB formation in both cell lines. In combination with RT, ID11 radiosensitized both cell lines, which correlated in both cases with a significant increase in initial DNA damage levels, yet the repair appeared effective.

Finally, ID12 was the second PEGylated GNP, and was assessed with 11 PEG groups in each PEG chain in the surface coating. In the absence of RT, our findings show that ID12 caused a small but significant decrease in cell survival only at 1 mM at both exposure times in U-87 MG cells, and at 24 h in U-251 MG cells. This difference in time-dependent effects seen between cell lines could potentially be due to variations in cellular uptake; however, additional experiments would need to be conducted. Similar to both the ID10 and ID11 DNA damage data, the observed ID12-mediated cytotoxicity did not correlate with significant changes in the DSB levels or cell cycle progression.

When combined with RT, ID12 caused slight radiosensitization in U-87 MG cells, less than ID11, and did not significantly alter DSB formation compared to the irradiated control. Longer PEG chains are inherently flexible, which can hinder binding with cell surface proteins on target cells, reducing cellular localisation and ultimately photoelectric absorption and radiosensitization as a consequence [15]. This may underlie the low ID12-mediated radiosensitization observed in U-87 MG cells only. In contrast, ID12 had the greatest radiosensitizing effect of all three GNPs in U-251 MG cells, alongside significantly increasing DNA damage yield, thus illustrating cell line-specific responses to ID12.

In this study, we observed SERs ranging from 1.04 to 2.08 which are broadly similar to the previously reported data for a range of different types of GNP formulations in different cell models [6]. It is important to note that our study was conducted using 225 kV X-ray, where the strong photoelectric absorption leads to dramatic increases in the absorbed dose that is not predicted based on the mass-energy attenuation coefficient of gold at that energy. A further limitation is that RT is delivered using megavoltage (MV) photons, which have energies typically ranging from 1 to 15 MeV in most clinical scenarios. At these energies, photon absorption in both gold and soft tissue is dominated by the Compton effect, which is weakly dependent on the Z of the absorbing material. Further studies at MV energies are required to validate our data.

The comparison of key radiological data demonstrated heterogeneous responses to the differentially PEGylated GNPs, indicating that PEG chain length plays a role in GNP therapeutic efficacy. PEGylation was shown to improve the radiosensitizing potential of GNPs in comparison to the unPEGylated counterpart, in agreement with our hypothesis. In the cases of ID11 and ID12, the observed radiosensitization correlated with significant DNA DSB induction, which may be an underlying mechanism of radiosensitization. As ID11 (four PEG groups) successfully radiosensitized both GBM cell lines, it may be considered to be the optimum GNP radiosensitizer of all three of the tested formulations.

Alongside radiosensitization analysis, this study also aimed to characterise the novel impact of the same three differentially PEGylated GNPs and RT on inflammatory cytokine expression in GBM cell lines. Considering the immune-avoiding abilities conferred by PEG to GNPs, as previously discussed, we hypothesized that the PEGylated GNPs ID11 and ID12, as monotherapies, would cause fewer changes in cytokine secretion compared to the unPEGylated GNP ID10. Our U-87 MG monotherapy findings support this hypothesis, with ID10 having a greater immunogenic effect with the most differentially expressed cytokines (proteins with a fold change of <0.5 and >2) identified compared to ID11 and ID12. While ID10 caused fewer differentially expressed cytokines in U-251 MG cells compared to ID11 and ID12, ID10 still caused the highest number of upregulated differentially expressed cytokines, suggesting a greater inflammatory response with ID10 treatment. Similar to the radiobiology data, this clearly demonstrates cell line-specific responses to each GNP used as monotherapies.

Contrary to monotherapy treatment, when ID11 was combined with 8 Gy RT, it achieved the highest number of differentially expressed cytokines. This indicates that ID11 enhanced the immunogenicity of RT in comparison to ID10 and ID12, suggesting that four PEG groups are optimal for generating a greater inflammatory response in GBM cells. This may have also contributed to the observed ID11-mediated radiosensitization in both cell lines. As previously mentioned, GBM is an extremely immune “cold” tumour, making ID11 an attractive candidate in novel RT-drug combinations for stimulating a greater anti-tumour immune response.

Pathway enrichment analysis of each cytokine profile provided a greater understanding of how these different GNPs and RT impact important biological processes. All GNP-RT combinations caused a universal upregulation of the interferon-γ response, alongside the associated IL-12, which is known to favourably activate anti-tumour macrophages of the M1 phenotype [30]. Alternatively, these same combinations simultaneously upregulated IL-4, which activates pro-tumour M2 macrophages, highlighting the complex interplay between immunosuppressive and immunostimulatory processes [31].

The cytokine MIP-1δ had the highest changes in expression across all GNP-RT combination treatments, which is normally minimally expressed by GBM despite being associated with disease progression [32,33,34]. This could provide a potential opportunity to target MIP-1δ in vivo to further the therapeutic efficacy of ID11 and ID12. Promisingly, both the PEGylated GNPs ID11 and ID12, in combination with RT, enhanced immune cell activation, including leukocytes and macrophages. Such infiltrating anti-tumour immune cells have been found to improve the therapeutic success of immunotherapies in GBM [35]. With particular relevance to GBM, the cytokines GCSF and GM-CSF were highly upregulated in response to ID12-RT treatment, both of which have been implicated in establishing and promoting the hostile immunosuppressive tumour microenvironment of GBM and tumour progression [36,37]. Thus, ID12-RT, in combination with GCSF or GM-CSF inhibitors, may also achieve improved radiobiological efficacy.

A deeper evaluation of the radiation response to each GNP, together with the corresponding cytokine expression analysis, revealed a correlation between radiosensitization and the extent of the upregulated cytokines which were identified. For example, in U-87 MG cells, ID11 caused the greatest sensitization with RT and simultaneously produced the highest number of upregulated differentially expressed cytokines, 22 in total. Again, in U-251 MG cells, ID12 was identified as the greatest GNP radiosensitizer which corresponded to the most upregulated differentially expressed cytokines, 11 in total. These findings demonstrate, for the first time, a correlation between elevated GNP efficacy as radiosensitizers and the strength of the pro-inflammatory response, which aligns with the already-established relationship between immunogenicity and therapeutic success of therapies.

Future research could focus on the quantification of GNP cellular uptake to provide greater insight into the potential relationship between the PEG chain length, GNP content within cells, and observed radiosensitization. The mass of gold per cell following transient treatment with each GNP may be quantified by inductively coupled plasma-optical emission spectroscopy (ICP-OES). The changes in the expression of highly differentially expressed cytokines could be validated by enzyme-linked immunosorbent assays (ELISAs). To better reflect the complexity of immune responses to these combination treatments, an in vitro co-culture model of GBM cells and THP1 immune cells could be used. This methodology is commonly implemented for investigating signalling pathways and macrophage activity in response to treatments [38]. In addition, in vivo evaluation with an immunocompetent model using CT-2A mouse GBM cells could provide greater insight into the translation of in vitro cytokine profiles to in vivo immune responses.

## 4. Materials and Methods

### 4.1. Synthesis and Physical Characterisation of GNPs (ID10, ID11 and ID12)

**Synthesis.** Most of the chemicals and solvents were purchased from Sigma-Aldrich Chimie S.a.r.l. (Saint-Quentin-Fallavier, France) and used without further purification. The chelators TADOTAGA, TAPEG_4_DOTAGA, and TAPEG_11_DOTAGA were obtained from CheMatech^®^ (Dijon, France) and used without further purification.

For the GNPs, 50 mg (1.22 × 10^–4^ mol) of HAuCl_4_•3H_2_O dissolved in 20 mL of methanol was placed in a 250 mL round-bottom flask. Then, 94, 115, or 153 mg (1.22 × 10^–4^ mol) of TADOTAGA, TAPEG_4_DOTAGA, or TAPEG_11_DOTAGA, respectively, in 10 mL of water, was added to the gold salt solution under stirring. The mixture turned from yellow to orange. After a few mins, 48 mg of NaBH_4_ dissolved in 3 mL of water was added to the mixture under vigorous stirring at room temperature. The stirring was maintained for 1 h. Then, the mixture was dialyzed using a 6000–8000 molecular-weight cut-off membrane. The dialysis tubing, which contains a crude colloidal suspension of GNPs, was immersed in a deionized water bath (the volume of the bath is equal to 20-fold the volume of the colloidal suspension). The bath was gently stirred and was changed twice a day for three days. After dialysis, the colloids were concentrated by centrifugation (Vivaspin^®^, 10 kDa, Sigma Aldrich, London, UK) at 1500 rpm until the gold concentration was approximately 50 mM.

**UV–visible spectroscopy.** UV–visible absorption spectra of functionalized GNP colloidal suspensions were recorded at room temperature with a SPECORD 250 spectrophotometer (Analytic Jena AG) in the 400–800 nm range. The spectral measurements were performed on a diluted colloid introduced in a standard quartz cuvette.

**Hydrodynamic diameter and zeta potential measurements.** Direct determination of the hydrodynamic diameter and zeta potential of nanoparticles was performed with a Zetasizer from Malvern Instrument. The suspension was diluted to obtain a concentration of 0.08 g/L in an aqueous solution (for hydrodynamic diameter measurements) and in NaCl (0.01 M) aqueous solution adjusted to the desired pH.

**Figure A3:**
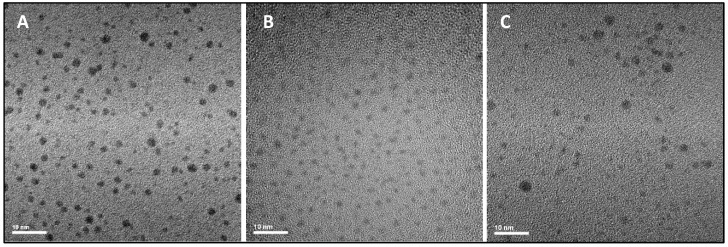
Transmission electron micrographs of (**A**) Au@TADOTAGA, (**B**) Au@TAPEG_4_DOTAGA, and (**C**) Au@TAPEG_11_DOTAGA.

**Transmission electron microscopy (TEM).** TEM was used to obtain detailed morphological information about the samples and was carried out using a JEOL 2010 microscope operating at 200 kV. The samples for TEM were prepared by depositing a drop of a diluted colloidal solution (Au@TADOTAGA, Au@TAPEG_4_DOTAGA, and Au@TAPEG_11_DOTAGA) on a carbon grid and allowing the liquid to dry in air at room temperature (Figure A3).

**Thermogravimetric analyses (TGA).** TGA was conducted to quantify the amount of DOTA chelators at the GNP surface. TGA was performed with a DISCOVERY device (TA Instruments), on ca. 2 mg of freeze-dried samples, under an air flow and a heating rate of 5 °C·min^–1^ in the temperature range 25–800 °C.

### 4.2. Cell Culture

The human GBM cell lines, U-87 MG, and U-251 MG were cultured in Dulbecco’s Modified Eagle Media (DMEM) (Sigma, USA) supplemented with 10% Foetal Bovine Serum (FBS) (Sigma) and 1% Penicillin Streptomycin (Pen-Strep) (Sigma). Cells were maintained in a humidified atmosphere at 37 °C with 5% CO_2_ and subcultured every 3–4 days to maintain exponential growth. All cell lines were acquired from the American Type Culture Collection (ATCC) and were routinely tested for mycoplasma. All cell lines were authenticated by ATCC using Short Tandem Repeat (STR) Screening.

### 4.3. Clonogenic Survival Assay, GNP Treatments, and In Vitro Irradiation Procedure

Cell survival was quantified by clonogenic assay following treatment with GNPs and in combination with radiation using the method described by Puck and Marcus [39]. Firstly, cells were seeded into 6-well plates at different densities depending on the dose of radiation delivered (0–2 Gy: 500 cells, 4 Gy: 1000 cells, and 8 Gy: 2000 cells). Cells were incubated for 24 h to adhere to the wells before treatment with GNPs at various concentrations (0.1–1 mM in 500 μL media per well) for either 1 h or 24 h prior to radiation. All concentrations of GNPs used in this study relate specifically to the concentration of gold. All cells were irradiated with 225 kVp X-rays generated using an X-Rad 225 generator (Precision X-ray Inc., CT, USA) with a 2 mm copper filter giving a half value layer (HVL) of 2.3 mm Cu. The mean energy of the X-ray spectrum was 113 kV and the peak energy occurred at around 100 kV. All quoted doses are the absorbed dose in water 50 cm from the radiation source at a dose rate of 0.591 Gy/min.

After irradiation, the GNP-containing media was aspirated and replaced with fresh media. Cells were incubated for 7–10 days depending on the cell line. Cells were then stained using crystal violet (0.4% in 95% ethanol) for 20 min before the excess was rinsed off. The number of colonies was scored using a 50-cell exclusion criterion and the surviving fraction (SF) of cells for each treatment was calculated as the proportion of colonies counted compared to the control cells. Survival curves were fitted to the LQ model of SF = exp (−αD−βD^2^) where D is the radiation dose (Gy), and α and β are the linear and quadratic components of cell killing, respectively. SERs were calculated as the ratio of the mean inactivation dose for control and NP-treated cells.

### 4.4. Analysis of DNA DSBs by Immunofluorescence Microscopy

DSBs were quantified by immunofluorescence staining of 53BP1 foci. Cells were seeded onto sterilised coverslips in 6-well plates (Starstedt, Germany) at a density of 1 × 10^5^ per well. Twenty-four hours later, GNPs were added at a concentration of 1 mM in 500 μL of media and cells were incubated for 1 h prior to irradiation at a dose of 2 Gy. Cells were fixed at 1 h and 24 h after irradiation in 50:50 methanol:acetone and permeabilised using methanol only. Cells were blocked with blocking buffer (5% FBS, 0.5% Triton X-100 in PBS) before being incubated with anti-53BP1 primary antibody (1:3000 in blocking buffer) for 1 h at room temperature. Cells were washed (0.1% Triton X-100 in PBS) and incubated with a secondary antibody (GAR 488, 1:2000 in blocking buffer) in the dark at room temperature for 1 h. Samples were stained using a DAPI antibody (1:20,000 in PBS), then coverslips were mounted onto glass slides using 20 μL ProLong Gold Antifade Mountant (Thermo Fisher Scientific, Waltham, MA, US). The number of DSB foci was counted for 50 randomly selected cells for each slide and averaged over 3 repeats.

### 4.5. Cell Cycle Analysis by Flow Cytometry

The proportion of cells within each phase of the cell cycle was analysed by flow cytometry. Cells were seeded into 6-well plates at a density of 1 × 10^5^ per well and allowed to adhere overnight. Cells were transiently treated with 1 mM GNPs for 1 h prior to 2 Gy irradiation. Samples were collected after 72 h and cells were detached by adding 2 mL 0.1% EDTA in PBS per well. Cells were centrifuged, washed (1 mL of 1% FBS in PBS), fixed (4 mL of ice-cold 100% ethanol), and then stored overnight at 4 °C until ready to stain. To stain with propidium iodide (PI), cells were centrifuged to remove excess ethanol and then resuspended with 6 mL of 1% FBS in PBS. Cells were centrifuged again, then cell pellets were resuspended with 500 µL staining solution (1% PI, 0.25% RNaseA in 1% FBS in PBS) and incubated at 37 °C for 30 min. Samples were analysed using the BD Accuri C6 Plus (BD Biosciences, UK) flow cytometer and the BD CSampler Plus software (V1.0.23.1). In total, 10,000 cells were analysed per sample and values were averaged over 2–3 repeats.

### 4.6. Human Cytokine Arrays and Bioinformatics Analysis

Cytokine arrays (ab133998, Abcam, Cambridge, UK) were performed to simultaneously characterise the protein expression of 80 human targets in response to GNPs and radiation treatment. Cells were seeded into P90 dishes at a density of 1 × 10^6^ per plate and incubated for 24 h to adhere. Cells were treated with 1 mM GNPs, 8 Gy radiation, or each GNP in combination with 8 Gy for 1 h, then treatments were replaced with fresh media. The cells and media were incubated for 72 h, then samples were collected.

The manufacturer’s instructions were followed when performing the arrays. Membranes were developed using the GBox Imager and Syngene software (V1.4.60). Quantification of signalling intensities was performed with GeneTools software (V4.3.14), and densitometry values for spots on treated membranes were normalised to that of the control membrane to allow for comparison.

Proteins which satisfied a threshold of >2- and <0.5- fold change were considered differentially expressed and selected proteins were transformed into their corresponding gene names. Analysis of enriched GO processes was performed using the online gene annotation resource Metascape (https://metascape.org/gp/index.html#/main/step1 (accessed on 15 January 2023). Venn diagrams were created using Venny (version 2.1.0). Cytokine arrays were performed in one independent experiment.

### 4.7. Statistical Analysis

Statistical analysis of the cytotoxicity and DNA damage bar graphs was performed by two-way ANOVA. Statistical analysis of the flow cytometry bar graphs was performed by one-way ANOVA. All data presented have been replicated in triplicate and in three independent experiments unless stated otherwise. All error bars represent the standard error mean (SEM). Statistical significances are represented as *p* < 0.05 = *; *p* < 0.01 = **; *p* <0.001 = ***; and *p* < 0.0001 = ****. All bar graphs, dose–response curves, heatmaps, and PCA plots were created using GraphPad Prism (version 9.2.0).

## 5. Conclusions

This study characterised, for the first time, both the radiosensitizing and immunomodulatory properties of GNPs with differential PEG chain lengths in combination with kVp X-rays in two GBM cell lines in vitro. Key radiobiological endpoints were evaluated, including clonogenic cell survival, DNA damage, and cell cycle distribution, alongside changes in cytokine expression profiles. The data strongly suggest that PEGylated GNPs have greater potential as radiosensitizers in comparison to their unPEGylated counterparts, with significant initial DNA damage induction as a possible underlying mechanism for sensitization. For radiosensitizing the GNPs ID11 and ID12, a higher number of upregulated inflammatory cytokines were also produced, suggesting a potential link between GNP radiosensitizing efficacy and a greater pro-inflammatory response. For enhancing both the therapeutic efficacy and immunogenicity of RT, these ultrasmall PEGylated GNPs are an attractive therapeutic option in the context of GBM and require further investigation.

## Figures and Tables

**Table 1:** Summary of radiosensitizing parameters of the differentially PEGylated GNPs in GBM cell models. This table describes the 𝛼/𝛽 ratios, SF_2_, and SER values for each GNP at 0.1–1 mM, with values taken from the corresponding linear quadratic curves in Figure 2 and Figure 3 for U-87 MG and U-251 MG cells, respectively, and for 1 h and 24 h exposure times.

		α/β		SF_2_		SER	
GNP	Conc	1 h	24 h	1 h	24 h	1 h	24 h
	(mM)						
U-87 MG							
**ID10**	**Control**	4.00	2.75	0.92	0.94		
	**0.1**	2.33	6.30	0.97	0.92	0.97	1.04
	**0.5**	2.90	8.59	0.98	0.89	0.99	1.08
	**1**	1.21	6.28	1	0.90	0.98	1.09
**ID11**	**Control**	8.37	6.78	0.80	0.78		
	**0.1**	4.40	2.37	0.86	0.79	0.99	0.94
	**0.5**	5.91	12.79	0.69	0.60	1.07	1.20
	**1**	4.56	14.95	0.55	0.47	1.21	1.45
**ID12**	**Control**	0	7.91	0.61	0.86		
	**0.1**	0	10.00	0.69	0.94	0.93	0.93
	**0.5**	0	39.07	0.54	0.81	1.10	1.00
	**1**	0	0	0.46	0.61	1.13	1.16
**U-251 MG**							
**ID10**	**Control**	0.94	33.39	1.01	0.73		
	**0.1**	2.24	5.69	0.92	0.86	1.04	0.92
	**0.5**	3.96	8.90	0.84	0.73	1.07	1.00
	**1**	0	3.83	0.85	0.74	0.96	0.95
**ID11**	**Control**	0	0	1.02	0.99		
	**0.1**	0	0	0.97	0.98	1.05	1.04
	**0.5**	0	0	1.13	0.94	0.95	1.08
	**1**	0	0	0.74	0.87	1.17	1.10
**ID12**	**Control**	4.42	16.44	0.86	0.72		
	**0.1**	0	11.06	1.2	0.57	0.85	1.15
	**0.5**	13.82	38.91	0.67	0.56	1.25	1.25
	**1**	0	0	0.37	0.37	2.08	1.70

## Data Availability

All data is available on request.

## Appendix A

**Table A1:** A summary of the GNP characteristics. These include the diameter of the gold core (d_core_), the hydrodynamic diameter (D_h_), the average number of macrocycles per NP (n_DOTAGA_), and zeta potential (ζ) at pH 7.4.

	Au@TADOTAGA (ID10)	Au@TAPEG_4_DOTAGA (ID11)	Au@TAPEG_11_DOTAGA (ID12)
**d_core_ (nm)**	2.5 ± 0.3	2.5 ± 0.3	2.5 ± 0.6
**D_h_ (nm)**	6.4 ± 2.3	6.4 ± 1.9	8.3 ± 1.7
**n_DOTAGA_**	85	71	87
**ζ (mV)**	−24.0	−33.9	−16.1
